# Practice Effects in Mild Cognitive Impairment Increase Reversion Rates and Delay Detection of New Impairments

**DOI:** 10.3389/fnagi.2022.847315

**Published:** 2022-04-25

**Authors:** Mark Sanderson-Cimino, Jeremy A. Elman, Xin M. Tu, Alden L. Gross, Matthew S. Panizzon, Daniel E. Gustavson, Mark W. Bondi, Emily C. Edmonds, Joel S. Eppig, Carol E. Franz, Amy J. Jak, Michael J. Lyons, Kelsey R. Thomas, McKenna E. Williams, William S. Kremen

**Affiliations:** ^1^University of California San Diego Joint Doctoral Program in Clinical Psychology, San Diego State University, San Diego, CA, United States; ^2^Center for Behavior Genetics of Aging, University of California, San Diego, San Diego, CA, United States; ^3^Department of Psychiatry, School of Medicine, University of California, San Diego, San Diego, CA, United States; ^4^Department of Family Medicine and Public Health, University of California, San Diego, San Diego, CA, United States; ^5^Sam and Rose Stein Institute for Research on Aging, University of California, San Diego, San Diego, CA, United States; ^6^Department of Epidemiology, Johns Hopkins Bloomberg School of Public Health, Baltimore, MA, United States; ^7^Department of Medicine, Vanderbilt University Medical Center, Nashville, TN, United States; ^8^Psychology Service, VA San Diego Healthcare System, San Diego, CA, United States; ^9^Research Service, VA San Diego Healthcare System, San Diego, CA, United States; ^10^Rehabilitation Institute of Washington, Seattle, WA, United States; ^11^Center of Excellence for Stress and Mental Health, Veterans Affairs San Diego Healthcare System, San Diego, CA, United States; ^12^Department of Psychological and Brain Sciences, Boston University, Boston, MA, United States

**Keywords:** practice effects, cognitive aging, mild cognitive impairment, Alzheimer’s disease, biomarkers, dementia progression

## Abstract

**Objective:**

Cognitive practice effects (PEs) can delay detection of progression from cognitively unimpaired to mild cognitive impairment (MCI). They also reduce diagnostic accuracy as suggested by biomarker positivity data. Even among those who decline, PEs can mask steeper declines by inflating cognitive scores. Within MCI samples, PEs may increase reversion rates and thus impede detection of further impairment. Within an MCI sample at baseline, we evaluated how PEs impact prevalence, reversion rates, and dementia progression after 1 year.

**Methods:**

We examined 329 baseline Alzheimer’s Disease Neuroimaging Initiative MCI participants (mean age = 73.1; *SD* = 7.4). We identified test-naïve participants who were demographically matched to returnees at their 1-year follow-up. Since the only major difference between groups was that one completed testing once and the other twice, comparison of scores in each group yielded PEs. PEs were subtracted from each test to yield PE-adjusted scores. Biomarkers included cerebrospinal fluid phosphorylated tau and amyloid beta. Cox proportional models predicted time until first dementia diagnosis using PE-unadjusted and PE-adjusted diagnoses.

**Results:**

Accounting for PEs increased MCI prevalence at follow-up by 9.2% (272 vs. 249 MCI), and reduced reversion to normal by 28.8% (57 vs. 80 reverters). PEs also increased stability of single-domain MCI by 12.0% (164 vs. 147). Compared to PE-unadjusted diagnoses, use of PE-adjusted follow-up diagnoses led to a twofold increase in hazard ratios for incident dementia. We classified individuals as false reverters if they reverted to cognitively unimpaired status based on PE-unadjusted scores, but remained classified as MCI cases after accounting for PEs. When amyloid and tau positivity were examined together, 72.2% of these false reverters were positive for at least one biomarker.

**Interpretation:**

Even when PEs are small, they can meaningfully change whether some individuals with MCI retain the diagnosis at a 1-year follow-up. Accounting for PEs resulted in increased MCI prevalence and altered stability/reversion rates. This improved diagnostic accuracy also increased the dementia-predicting ability of MCI diagnoses.

## Introduction

### Mild Cognitive Impairment Stability and Reversion

Mild cognitive impairment (MCI) is characterized by cognitive deficits in the presence of minimal to no impairment in functional activities (Manly et al., 2008; Albert et al., 2011). MCI is seen as a risk factor for Alzheimer’s Disease dementia (AD), particularly when there is a memory impairment either alone (i.e., single-domain amnestic MCI) or in combination with deficits in other domains (i.e., multi-domain amnestic MCI) (Manly et al., 2008; Albert et al., 2011; Eppig et al., 2020; Thomas et al., 2020). Individuals diagnosed with MCI are significantly more likely to progress to AD, and do so at a faster rate than those without MCI (Mitchell and Shiri-Feshki, 2009; Pandya et al., 2016). Individuals with MCI who are on the AD trajectory often have AD biomarker levels in between those diagnosed as cognitively normal (CN) and those with AD (Edmonds et al., 2015a; Olsson et al., 2016).

Nearly all AD clinical trials have focused on treating individuals with dementia in an effort to mitigate or reverse the disease. Unfortunately, the failure rate for these trials is greater than 99% (Cummings et al., 2014; Anand et al., 2017). As a result, there has been a shift toward identifying and targeting individuals at the earliest stages of the disease including at-risk CN and MCI (Sperling R. et al., 2014; Sperling R. A. et al., 2014; Canevelli et al., 2016; Anand et al., 2017; Alexander et al., 2021). As noted by Canevelli et al. (2016), at least 274 randomized controlled trials were recruiting MCI subjects in 2016. As such, accurate diagnoses of earlier disease stages are necessary to further the treatment of AD (Edmonds et al., 2018; Veitch et al., 2019; Eppig et al., 2020).

There is concern regarding stability of MCI diagnosis that limits its use in clinical and research settings. Although 10–12% of those with MCI are expected to convert to AD per year, 20–50% of individuals revert from MCI to CN status within 2–5 years (Pandya et al., 2016). Over a similar time frame, an estimated 37–67% of individuals retain their MCI diagnosis (Pandya et al., 2016). One criticism of the MCI diagnosis has centered on the fact that individuals are more likely to revert to CN or maintain their MCI status than to convert to dementia each year (Canevelli et al., 2016). On the other hand, long term follow-ups may be necessary to more accurately determine the true proportion of those with MCI who progress to dementia.

Much of the MCI reversion rate literature was published prior to 2016 and was summarized by three articles (Canevelli et al., 2016; Malek-Ahmadi, 2016; Pandya et al., 2016). These authors highlighted the wide range in reversion rates and suggested that this variability is likely due to multiple factors, including the heterogeneity of MCI criteria and reversible causes such as depression (Canevelli et al., 2016; Malek-Ahmadi, 2016; Pandya et al., 2016). Malek-Ahmadi (2016) and Pandya et al. (2016) also suggested that reducing reversion rates should be an essential goal of future MCI methodology studies. Canevelli et al. (2016) and Pandya et al. (2016) argued that MCI may be an unstable condition where reversion to normal is expected, and that its use as a prodromal stage of underlying neurodegenerative diseases is questionable. Malek-Ahmadi (2016) suggested that the utility of MCI diagnosis would benefit from further refinement of statistical methods, the use of sensitive cognitive tests, and greater utilization of biomarkers. All three reviews concluded that reversion impairs our ability to treat AD by diluting samples and reducing study power (Canevelli et al., 2016; Malek-Ahmadi, 2016; Pandya et al., 2016).

### Practice Effects and Mild Cognitive Impairment

Practice effects (PEs) on cognitive tests used to diagnose MCI are a likely contributor to MCI reversion rates. They mask cognitive decline by increasing scores at follow-up testing relative to how an individual would have performed if they were naïve to the test. PEs are due to familiarity with specific test items (i.e., content effect), and/or increased comfort and familiarity with the general assessment process (i.e., context effect) (Calamia et al., 2012; Gross et al., 2017). PEs in participants without dementia have been found across retest intervals as long as 7 years, and across multiple cognitive domains (Ronnlund et al., 2005; Gross et al., 2015; Elman et al., 2018; Wang et al., 2020). PEs after 3–6 months have even been observed in those with mild AD who performed very poorly on memory measures (Goldberg et al., 2015; Gross et al., 2017). Although PEs may be small in cognitively impaired samples, we have previously shown that utilizing that information to change MCI classification increases diagnosis accuracy and leads to earlier detection of decline (Goldberg et al., 2015; Jutten et al., 2020; Sanderson-Cimino et al., 2020).

The MCI classification methods, particularly in research, almost always rely on use of cut-off scores to define cognitive impairment (Winblad et al., 2004; Jak et al., 2009). The same cut-off is typically applied at baseline and follow-up visits. If an individual with MCI at baseline experiences a PE greater than their cognitive decline, then they may be pushed above the threshold for impairment despite having no change or even a slight decline in their actual cognitive ability. Even if there was no change in cognitive capacity, this individual would likely be misclassified as CN at follow-up, appearing to revert when in fact they still have MCI. The impact of PEs on MCI reversion rates has not been explicitly studied, but it is often suggested when reversion rates are discussed (Malek-Ahmadi, 2016; Thomas et al., 2020).

### Present Study

In the present analyses, we utilized a sample of Alzheimer’s Disease Neuroimaging Initiative (ADNI) participants who were diagnosed as MCI at baseline. We sought to (1) calculate 1-year follow-up cognitive classifications using PE-unadjusted and PE-adjusted scores, (2) compare reversion rates and diagnostic stability between PE-unadjusted and PE-adjusted classifications, and (3) provide criterion validity for the PE-adjusted classifications through baseline biomarker data and time until first dementia diagnosis. We hypothesized that the PE-adjusted scores would reveal false reverters, i.e., participants at follow-up who were classified as CN *via* PE-unadjusted scores but MCI *via* PE-adjusted scores. By retaining these participants in the MCI pool, we expected the PE-adjusted classifications to result in improved diagnostic stability and decreased reversion rates. Also, we expected the biomarker profile and the time until first dementia diagnosis of the false reverters to be more similar to the stable MCI participants than to true reverters (i.e., individuals classified as CN at follow-up based on both PE-adjusted and PE-unadjusted scores). Finally, in a *post hoc* analysis, we modeled the impact of PE adjustment on studies concerned with progression to dementia, a common outcome in clinical drug trials and research studies.

## Materials and Methods

### Participants

Data used in the preparation of this article were obtained from ADNI^1^. The ADNI, led by Principal Investigator Michael W. Weiner, MD, was launched in 2003 as a public-private partnership. The primary goal of ADNI has been to test whether serial magnetic resonance imaging, positron emission tomography, other biological markers, and clinical and neuropsychological assessment can be combined to measure the progression of MCI and early AD. For up-to-date information, see www.adni-info.org. Participants from the ADNI-1, ADNI-GO, and ADNI-2 cohorts were included.

Mild cognitive impairment was diagnosed using the Jak-Bondi approach (Jak et al., 2009; Bondi et al., 2014; Edmonds et al., 2018). Participants were classified as single domain MCI (amnestic, dysexecutive, or language-impaired) if their scores on 2 tests within the same cognitive domain were both greater than 1 SD below normative means. They were diagnosed as multi-domain MCI if they met the criteria for single domain MCI in more than one cognitive domain (e.g., impaired on both memory tasks and language tasks). The Jak-Bondi approach to MCI classification is favorable when compared with Petersen criteria with regard to the likelihood of progression to dementia, reversion rates, and proportion of biomarker-positive cases (Bondi et al., 2014; Edmonds et al., 2018).

We identified 344 individuals who were classified as MCI at baseline. Of those 344, 329 returned for a 12-month follow-up visit and also completed all cognitive measures at both assessments. Mean educational level of returnees was 16.4 years (*SD* = 2.9), 61.4% (*n* = 202) were female, and mean baseline age was 73.1 years (*SD* = 7.4).

### Procedures

Six cognitive tests were examined across the approximately 12-month test–retest interval. Episodic memory tasks included the Wechsler Memory Scaled-Revised, Logical Memory Story A delayed recall, and the Rey Auditory Verbal Learning Test (AVLT) delayed recall. Language tasks included the Boston Naming Test and Animal Fluency. Attention-executive function tasks were Trails A and Trails B. The American National Adult Reading Test provided an estimate of premorbid IQ. Only participants who had complete test data and completed the same version of tests at the baseline and 12-month visits were included.

*Z*-scores were calculated for the PE-adjusted and -unadjusted scores based on independent external norms that accounted for age, sex, and education for all tests except the AVLT (Shirk et al., 2011). The AVLT was *z*-scored based on the ADNI participants who were CN at baseline (*n* = 889) because we were unable to find appropriate external norms for this sample that also accounted for age, sex, and education. AVLT demographic corrections were based on a regression model that followed the same approach as the other normative adjustments. Beta values were multiplied by an individual’s corresponding age, sex, and education. The products were then removed from the AVLT raw scores. These adjusted AVLT scores were then *z*-scored.

Baseline biomarkers included cerebrospinal fluid amyloid-beta (Aβ), phosphorylated tau (p-tau), and total tau (t-tau). The ADNI biomarker core (University of Pennsylvania) used the fully automated Elecsys immunoassay (Roche Diagnostics). Sample collection and processing have been described previously (Shaw et al., 2009). Cutoffs for biomarker positivity were^2^ : Aβ+: Aβ < 977 pg/mL; p-tau+: p-tau > 21.8 pg/mL; t-tau+: t-tau > 270 pg/mL (Hansson et al., 2018; Elman et al., 2020). There were 226 returnees with biomarker data.

Dementia was diagnosed according to ADNI criteria: (1) Memory complaint by subject or study partner that is verified by a study partner; (2) Mini-Mental State Examination score between 20–26 (inclusive); (3) Clinical Dementia Rating score of either 0.5 or 1; (4) An impaired delayed memory score on the Logical memory test: ≤ to 8 for 16 or more years of education; ≤ to 4 for 8–15 years of education; or ≤to 2 for 0–7 more years of education; (5) National Institute of Neurological and Communicative Disorders and Stroke–Alzheimer’s Disease and Related Disorders Association criteria for probable AD (Petersen et al., 2010). No participants met these criteria at baseline or at the 12-month follow-up.

### Replacement-Participants Approach to Practice Effects

Although review papers have noted that PEs can exist even when there is longitudinal decline in observed performance, as expected within a sample at risk for AD (Salthouse, 2010), few have empirically demonstrated that claim (Goldberg et al., 2015). In such situations, Calamia et al. (2012) suggested that the most suitable approach is to utilize replacement participants (Rönnlund and Nilsson, 2006). To our knowledge, the replacement-participant approach has only been utilized in two samples (Ronnlund et al., 2005; Elman et al., 2018). In this method new participants are recruited for testing at follow-up who are demographically matched to returnees. The only difference between the groups is that replacements are taking the tests for the first time whereas returnees are retaking the tests. As age is one of the matching factors, any age-related decline should be equal across the groups. Therefore, comparing scores at follow-up between returnees and replacement participants (with additional adjustment for attrition effects) allows for detection of PEs when observed scores remain stable and—unlike other methods—even when they decline. In both scenarios, scores would have been lower without repeated exposure to the tests (Ronnlund et al., 2005; Elman et al., 2018).

The goal of the replacement method is to obtain follow-up scores at retest that are free of PEs and comparable to normative data (which assume no presence of PEs). Some researchers have used PEs in other ways, such in short-term retest paradigms (Duff et al., 2011, 2014; Duff, 2014; Duff and Hammers, 2020). The goal of this approach is to predict future decline and the likelihood of progressing to MCI or dementia (Jutten et al., 2020). Rather than predict decline, the goals of the replacement method are: (1) to detect decline at a given point in time that has been masked due to PEs, and (2) to revise the diagnosis of CN or MCI based on cognitive scores that have been appropriately adjusted to reflect the estimated magnitude of masked decline. Furthermore, only the replacement method has been empirically shown to calculate PEs when there is observable decline over time (Calamia et al., 2012; Elman et al., 2018). This attribute of the method makes it uniquely appropriate for samples that are impaired at baseline and/or are expected to decline over time (Calamia et al., 2012). Also, unique to this method is the fact that it allows for a change in how early MCI may be diagnosed.

### Practice Effect Calculation

Because replacement participants were not part of the original ADNI study design, we created what we refer to as the pseudo-replacement method of PE adjustment. We have fully described this method previously in an examination of individuals who were cognitively normal at baseline (Sanderson-Cimino et al., 2020). Briefly, a bootstrap approach (5,000 resamples, with replacement) was used to calculate PE values for each cognitive test. At every bootstrap iteration, a subsample of returnees was randomly selected (25% of sample) from the total number of individuals who had a baseline and 12-month follow-up visit. We then removed these selected returnees from the overall baseline pool, leaving a subset of potential “pseudo-replacement participants” that included returnees not chosen at that iteration and those who did not return for a follow-up (approximately 75% of the sample). From this potential replacement pool, a set of pseudo-replacements was matched to selected returnees on age at returnee follow-up, sex, years of education, and premorbid IQ using one-to-one matching and propensity scores (R package: MatchIt) (Ho et al., 2018). Additional *t*-tests and chi-squared tests ensured that returnees and pseudo-replacements were matched at a group level (*p*s > 0.8). Thus, this sample of pseudo-replacement participants was demographically identical to the returnee subsample. In a traditional replacement participants method of PE-adjustment returnees and non-returnees are combined into a “baseline” subsample that excludes replacements. In this method, we used a “proportional baseline” subsample that included the baseline scores for the returnees chosen at that iteration as well as all other subjects not chosen to be pseudo-replacements (approximately 75% of sample). However, the removal of the pseudo-replacements from the sample led to an artificially high portion of lower-performing baseline participants since the pseudo-replacements perform at a similar level to returnees at baseline. To correct for this issue, we calculated the retention and attrition rates for that visit in the overall sample. Because the PE for each test was calculated individually, we used test-specific retention and attrition rates, which resulted in a slight variation in rates; the average retention rate was 66% (65–70%) and the average attrition rate was 34% (30–35%). We then used these rates in the creation of the proportional baseline mean (see below). Of note, due to the bootstrapping and matching procedure, the number of participants in each group (i.e., returnees and replacements) varied but was always greater than 80 participants.

The equations below were used to calculate the PE:


Differencescore=Returnees-T2Pseudo-Replacements1T

Attritioneffect=Returnees-T1ProportionalBaseline1T

Practiceeffect=Differencescore-AttritionEffect

Where Returnees_*T*2_ represents the mean score of the returnee sample at their second assessment, Pseudo-replacements_*T*1_ represents the mean score of the pseudo-replacement sample (by definition, at their first assessment), and Returnees_*T*1_ represents the mean score of returnees at their first assessment. The Proportional Baseline_*T*1_ was a weighted mean calculated by multiplying the returnee baseline scores by the test-specific retention rate (65–75%) and the remaining portion of the subsample by the test-specific attrition rate (30–35%%). The difference score represents the sum of the PE and the attrition effect. The attrition effect accounts for the fact that individuals who return for follow-up are typically higher-performing or healthier than those who drop out. Subtracting the attrition effect from the difference score prevents over-estimation of the PE (Ronnlund et al., 2005; Elman et al., 2018). Use of a proportional baseline that retains the test-specific retention and attrition rates prevents overestimation of the attrition effect as removing the pseudo-replacements from this sample artificially lowers the baseline mean score. The PE for each test was calculated by subtracting the attrition effect from the difference score.

### Statistical Analysis

After calculation, the PE for each test was then subtracted from each individual’s observed (unadjusted) follow-up test score to provide PE-adjusted raw scores. Cohen’s *d* was calculated for each PE by comparing PE-unadjusted and PE-adjusted scores. Adjusted raw scores at follow-up were converted to *z*-scores, which were used to determine PE-adjusted diagnoses. Stated differently, a score was labeled as impaired if the follow-up PE-adjusted score was greater than 1 SD below the average demographic-corrected mean. To evaluate the impact PE-adjustment had on cognitive classification, McNemar χ^2^ tests were used to compare differences in the proportion of individuals classified as having MCI before and after adjusting for PEs. To assess criterion validity of the PE-adjusted diagnoses, McNemar χ^2^ tests were used to compare the number of biomarker-negative reverters and biomarker-positive stable MCI participants when using PE-adjusted versus PE-unadjusted scores.

Time until first dementia diagnosis in months from baseline was also used to validate PE-adjusted diagnoses. Cognitive data used to diagnose dementia by ADNI were not adjusted for PEs. Wilcoxon rank sum tests were used to compare groups due to the non-normal distribution of months until first dementia diagnosis. It was expected that those who reverted to CN status at follow-up would progress to dementia more slowly than those who remained classified as having MCI. As such, if PE adjustment improved diagnostic accuracy by correctly relabeling some false reverter (based on PE-unadjusted scores) as MCI, then a comparison between MCI and CN groups should show a larger and more statistically significant difference when using PE-adjusted scores than when using PE-unadjusted scores. PE-adjustment should also alter a comparison between those who truly revert and the false reverters, with false reverters progressing faster than true reverters. The following four time-until-dementia comparisons were tested: PE-adjusted MCI versus PE-adjusted CN; PE-unadjusted MCI versus PE-unadjusted CN; False reverters versus PE-unadjusted MCI; and False reverters versus PE-adjusted CN.

We also expected that the false reverters (based on PE-unadjusted scores) would have a biomarker profile more similar to the stable MCI participants than the true reverters. Thus, we calculated rates of biomarker positivity for diagnostic groups (Stable MCI and reverters) first using PE-unadjusted scores and then with PE-adjusted scores.

In *post hoc* analyses, Cox proportional hazard models compared progression to dementia between those who were diagnosed as MCI at follow-up and those who reverted to CN. All models used classification (Stable MCI vs. reverters) as the independent variable of interest and months from baseline until first dementia diagnosis as the dependent variable. Covariates were age and education. Models were completed first with PE-unadjusted scores and then with PE-adjusted scores.

Time-to-dementia analyses included a full model and three timeframe-restricted models: 16–150 months (full sample data), 16–24, 16–36, and 16–48 months. The models with restricted timeframes attempted to demonstrate how predictive the classification was for studies with shorter follow-up periods. Because, in these hypothetical studies, we could not know if a participant progressed to dementia past the specified timeframe, each model was right-censored with time to event defined as time to first dementia diagnosis or time to last follow-up within the restricted time period. As this project utilized existing data, the maximum follow-up period was set to 150 months because that was the longest available timeframe within ADNI.

## Results

PEs were non-zero for 5 of the 6 measures (Table 1) and ranged in magnitude (Cohen’s *d* = 0.06–0.26). PE-adjustment resulted in 23 more participants (+9%) classified as MCI at 1-year follow-up than when using PE-unadjusted scores (272 vs. 249). Of the 23, 16 (+9%) were classified as single-domain MCI and 7 participants classified as multi-domain MCI (+9%). Regarding specific cognitive domains, PE-adjustment resulted in 24 more participants (+11%) classified with memory impairment (233 vs. 209), 6 more participants (+9%) classified with attention-executive impairments (73 vs. 67), and 5 more participants (+7%) classified with language impairments (72 vs. 67). Full results are presented in Table 2.

**TABLE 1A:** Descriptive statistics among participants at baseline and 1-year-follow-up.

	Memory		Attention/executive function		Language	
Raw mean score (*SD*)	RAVLT	Logical memory	Trails A	Trails B	Boston naming	Category fluency
Full sample baseline	1.55 (2.61)	5.81 (3.57)	39.27 (20.85)	106.14 (66.90)	27.82 (3.76)	15.88 (4.76)
Full sample follow-up	2.17 (3.09)	6.39 (4.55)	39.39 (20.67)	106.44 (74.67)	28.15 (4.10)	15.29 (5.51)

*The “Full Sample” rows refer to the means (standard deviations) of all participants at baseline and at follow-up.*

**TABLE 1B:** Descriptive statistics and calculated practice effects for tests among participants classified as mild cognitive impairment at baseline.

	Memory		Attention/executive function		Language	
Raw mean score (*SD*)	RAVLT	Logical memory	Trails A	Trails B	Boston naming	Category fluency
Proportional baseline	1.59 (2.61)	1.92 (3.68)	40.28 (22.75)	109.76 (75.03)	27.66 (4.16)	15.51 (4.82)
Returnees baseline	1.58 (2.61)	2.00 (3.56)	39.88 (21.73)	107.45 (68.16)	27.77 (3.94)	15.70 (4.81)
Returnees follow-up	2.45 (3.07)	2.84 (4.51)	39.30 (22.19)	107.73 (76.53)	28.11 (4.51)	15.02 (5.46)
Replacements follow-up	1.67 (2.57)	1.86 (3.72)	41.35 (22.63)	114.40 (74.90)	27.37 (4.51)	15.11 (4.81)
Attrition effect	−0.01 [−0.13, 0.16]	0.09 [−0.10, 0.43]	−0.40 [−1.57, 0.89]	−2.31 [−6.64, 2.27]	0.11 [−0.14, 0.33]	0.43 [0.15, 0.72]
Practice effect	0.80 [−0.33, 3.08]	0.89 [−0.41, 3.33]	−1.64 [−5.65, 2.41]	−4.36 [−19.16, 9.57]	0.63 [−0.21, 1.53]	NA
Cohen’s *d*	0.26	0.20	−0.07	−0.06	0.14	NA

*Groups are based on the average performance across all 5,000 bootstrapped iterations. Means are based on transformed data that was reverted back to raw units. “Proportional baseline” refers to a weighted mean that combines the returnee baseline group and a group that included all subjects not selected to be Returnees or Replacements in that bootstrapped iteration. “Returnee Baseline” refers to baseline test scores for the subset of participants who returned for the 12-month follow-up visit (ns > 80) and were selected at that iteration. “Returnee Follow-Up” refers to 12-month scores for the same subset of returnees who were selected for that iteration. “Replacement Follow-up” refers to the pseudo-replacement scores (ns > 80). The scores for memory tasks indicate the number of words remembered at the delayed recall trials. Scores on the attention/executive functioning tests indicate time to completion of task. On these tasks, higher scores indicate worse performance. Scores on the Boston Naming Task indicate number of correct items identified; scores on Category Fluency indicate number of items correctly stated. Practice effects and attrition effects are in raw units with the 2.5 and 97.5 percentiles in brackets. As such, the negative practice effects and attrition effects for the Trails tasks demonstrates that practice decreased time (increased performance). Cohen’s d is given for the difference between PE-adjusted and unadjusted scores of returnees at follow-up. RAVLT, Rey Auditory Verbal Learning Test.*

**TABLE 2:** Classification prevalence at baseline and follow-up.

	Any MCI	M MCI	S MCI	Memory impairment	Attention/EF impairment	Language impairment	CN
Baseline	329	75	254	267	77	70	0
Unadjusted	249	79	170	209	67	67	80
Adjusted	272	86	186	233	73	72	57
Difference	+23	+7	+16	+24	+6	+5	−23
% difference	9.23%	8.86%	9.41%	11.48%	9.00%	7.46%	28.75%
χ^2^; *p*-value	21.0; *p* < 0.001	5.1; *p* = 0.02	7.5; *p* = 0.006	22.0; *P* < 0.001	3.2; *p* = 0.07	3.2; *p* = 0.07	21.0; *p* < 0.001

*Presents the number of participants who met criteria for mild cognitive impairment (MCI). The “unadjusted” and “adjusted” rows refer to diagnoses at the follow-up visit. The “Any MCI” column presents the count of participants who meet criteria for MCI in any domain, combining those who are impaired in only one domain (single-domain MCI: S MCI) and those who are impaired in 2 or 3 domains (multiple-domain MCI: M MCI). The impairment columns present the count of participants who were impaired in each domain, regardless of whether they are impaired in another domain. Individuals who do not meet criteria for impairment (i.e., classified as Cognitively Normal; CN) are displayed in the “CN” column.*

*The Difference row displays how many more participants meet criteria for that classification or impairment when adjusting for practice effects (i.e., Adjusted count – Unadjusted count). The percent listed in this row displays the percent increase/decrease when accounting for practice effects: difference/Unadjusted count. McNemar χ^2^ tests were used to evaluate the impact of practice-effect adjustment on classification or impairment count; p-values are presented.*

The overall 1-year stability of MCI (lack of reversion to CN) was raised by 7% when adjusting for PEs (PE-adjusted stability rate = 82.7%; PE-unadjusted stability rate = 75.6%). Across groups (single-domain MCI, multi-domain MCI) and within each cognitive domain (memory, attention-executive, and language), PE adjustment increased the number of participants who retained their baseline diagnosis of MCI (Range: +2 [+3%] to +22 [+11%]). In particular, there were significantly more participants who remained in the impaired range at follow-up on memory when using PE-adjusted data versus PE-unadjusted data (+11%; 201 vs. 223). A similar significant result was also found when considering stability of single-domain MCI (+12%; 147 vs. 164). Table 3 provides full stability results.

**TABLE 3:** Impact of practice effects on classification stability and progression.

	Stable M MCI	Stable S MCI	Progression to M MCI	Stable impairment		
				Memory	Attention/EF	Language
Unadjusted	45	147	34	201	46	42
Adjusted	49	164	37	223	48	44
Difference	+4	+17	+3	+22	+2	+2
% difference	8.89%	11.56%	8.82%	10.94%	4.35%	4.76%
χ^2^; *p*-value	2.25; *p* = 0.13	11.13; *p* < 0.001	1.3; *p* = 0.25	20.0; *p* < 0.001	0.5; *p* = 0.48	0.5; *p* = 0.48

*Displays the number of individuals classified as impaired at follow-up via practice effect-unadjusted scores and -adjusted scores. The “Stable M MCI” column provides the count of participants who met criteria for multiple domain mild cognitive impairment (M MCI) at baseline and at follow-up. The “Stable S MCI” provides the same information about individuals with single domain MCI (S MCI). Individuals who progressed from S MCI at baseline to M MCI at follow-up are displayed in the “Progression” column. The “Stable Impairment” section describes the number of individuals who retained an impairment in a specific cognitive domain at follow-up, regardless of whether they met criteria for an impairment in another domain at either visit. The Difference row displays how many more participants meet criteria for that classification or impairment when adjusting for practice effects (i.e., Adjusted count – Unadjusted count). The percent listed in this row displays the percent increase in stability when accounting for practice effects: difference/Unadjusted count. McNemar χ^2^ tests were used to evaluate the impact of practice-effect adjustment on classification or impairment stability; p-values are presented.*

The overall reversion rate (i.e., being classified as CN at follow-up) was 24.3% (*n* = 80) using PE-unadjusted scores and 17.3% (*n* = 57) using PE-adjusted scores. This indicates that adjusting for PEs resulted in a 28.8% reduction in the overall reversion rate. Table 4 describes how PE adjustment affects reversion rates across diagnostic subgroups and cognitive domains. Among those with single-domain MCI at baseline, adjusting for PEs reduced reversion rates by 27.4% (53 vs. 73 reverters). Regarding specific cognitive domains, adjustment reduced the reversion rate among those with baseline memory impairments by 33.3% (44 vs. 66). Adjustment also decreased reversion rates among the remaining cognitive domains (attention-executive and language) as well as among those who were multi-domain MCI at baseline (reversion to CN rate reduction range: 6.5–13.3%), but this equated to only a small change in the number of participants (ns < 5).

**TABLE 4:** Practice effect-adjustment and reversion rates.

	Reverters M MCI	Reverters S MCI	Reversion in specific domain		
			Memory	Attention/EF	Language
Count					
Unadjusted	30	73	66	28	31
Adjusted	26	53	44	26	29
Difference	−4	−20	−22	−2	−2
χ^2^; *p*-value	2.25 *p* = 0.13	18.1 *p* < 0.001	20.0 *p* < 0.001	0.5 *p* = 0.48	0.5 *p* = 0.48
Reversion rate					
Unadjusted	40.5%	28.7%	24.7%	36.3%	44.3%
Adjusted	35.1%	20.9%	16.5%	33.8%	41.4%
Difference	−5.4%	−7.8%	−8.2%	2.6%	2.9%
% change in reversion	Δ13.3%	Δ27.4%	Δ33.3%	Δ7.1%	Δ6.5%

*The “Count” section displays the number of participants who reverted from a classification or impairment based on practice effect-unadjusted and -adjusted data. Those who reverted from multi-domain mild cognitive impairment (M MCI) at baseline to either single domain MCI (S MCI) or cognitively normal are displayed in the “Reverters M MCI” column. Those who were classified as S MCI at baseline and reverted to cognitively normal at follow-up are listed in the “Reverters S MCI” column. The “Reversion in Specific Domain” section refers to individuals who had a baseline impairment in a domain (memory, attention/executive functioning, or language) but not at follow-up; participants in these columns may be impaired in other domains at either baseline or follow-up. The Difference row displays how many fewer participants reverted when adjusting for practice effects (i.e., Adjusted count – Unadjusted count). McNemar χ^2^ tests were used to evaluate the impact of practice-effect adjustment on classification or impairment reversion; p-values are presented.*

*The “Reversion Rate’ section lists the reversion percent for each column by dividing the counts provided above by the baseline prevalence of each classification shown in Table 1. For example, 74 people were classified as M MCI at baseline and 30 reverted at follow-up when using unadjusted data. Therefore, the reversion rate for the unadjusted M MCI reverters was 30/74. The difference row subtracts the reversion rate using Unadjusted data from the rate using Adjusted data. The “% change in reversion” row shows the percent change in reversion rate by dividing the difference by the unadjusted reversion rate: e.g., Δ13.3 = 5.4/40.5.*

We also compared how PE-adjusted and PE-unadjusted classification affected rate of progression to dementia. Of the 329 returnees, 159 progressed to dementia (48% of sample). As shown in Table 5, those who were diagnosed as MCI at follow-up and progressed to dementia during the study were first diagnosed in approximately the same time frame, regardless of PE consideration (median = 25.0 months). Those who reverted to CN and later progressed to dementia did so more slowly than the stable MCI groups (PE-unadjusted median = 37.3 months; PE-adjusted median = 60.3 months). In PE-unadjusted groups, based on Mann–Whitney *U* tests, there was no significant difference in time until first dementia diagnosis between stable MCI and reverter participants (*W* = 1703; *p* = 0.177). However, in the same comparison based on PE-adjusted scores, those in the stable MCI group progressed significantly faster than those who reverted to CN (*W* = 1240; *p* = 0.017).

**TABLE 5:** Progression to dementia.

	Full sample *N* = 159	Stable MCI		Reverters		False reverters *N* = 10
Months until DX		Unadjusted *N* = 141	Adjusted *N* = 151	Unadjusted *N* = 18	Adjusted *N* = 8	
Mean	37.48	36.17	36.32	47.77	59.44	38.44
Median	25.28	24.98	24.98	37.28	60.28	30.03
*SD*	21.90	20.66	20.66	28.68	33.34	21.70

*Presents the time in months until first dementia diagnosis (DX) among those who converted to dementia. Of the 329 participants 159 have progressed to dementia (“Full Sample”). Participants were classified as “Stable MCI” if they retained their mild cognitive impairment (MCI) classification at follow-up; participants were classified as “Reverters” if they were classified as cognitively normal at follow-up. Classifications were made using practice effect-unadjusted (“Unadjusted”) and practice effect-adjusted (“Adjusted”) data. Those who were classified as MCI by the practice effect-adjusted data but not the unadjusted data are referred to as “False reverters”. Values are bolded to emphasize that the False reverters appear to be similar to the Stable MCI group in time to first dementia diagnosis.*

Ten of the false reverters (6.2%) progressed to dementia. These participants progressed to dementia in a similar time frame as the those diagnosed with MCI *via* PE-unadjusted scores (median = 30.03 months). The false reverters progressed to dementia more quickly than those who were classified as CN based on PE-adjusted scores at follow-up. There was not a significantly different rate of progression to dementia between false reverters and PE-adjusted CNs, or between false reverters and PE-unadjusted MCI based on Mann–Whitney *U* tests (*p*s > 0.17).

**FIGURE 1:**
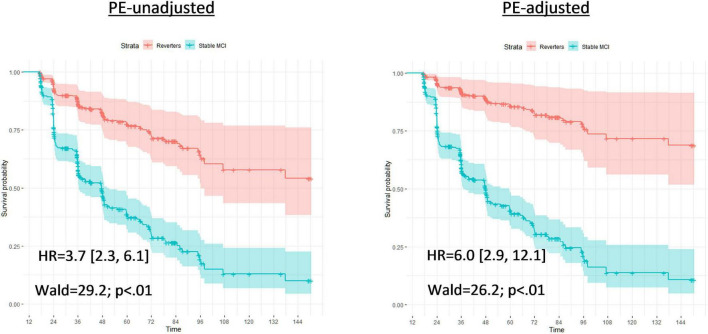
Full Cox proportional models for time until first dementia diagnosis by PE-unadjusted and PE-adjusted 12-month diagnoses. Cox proportional hazard models compared progression to dementia between those who were classified with mild cognitive impairment at follow-up (Stable MCI) and those who reverted to cognitively normal (Reverters). Models used classifications (Stable MCI vs. Reverter) as the independent variable of interest; months from baseline until first dementia diagnosis as the dependent variable; and all variable data (16 – months from baseline). Covariates were age and education, fixed at the average level within the sample (age: 73.1 years; education: 16.4 years). The left graph bases diagnoses on the PE-unadjusted 12-month data; the right graph uses diagnoses based on the PE-adjusted 12-month data. Each model presents a hazard ratio (HR; [CI]) that indicates how much more likely the Stable MCI group was to convert to dementia compared to the Reverters. Wald tests and likelihood-ratio tests (LRT) are also included with associated *p*-values to denote the significance of the HR. The *Y*-axis of each model provides the survival probability and the *X*-axis of each model provides the time frame until dementia conversion.

**FIGURE 2:**
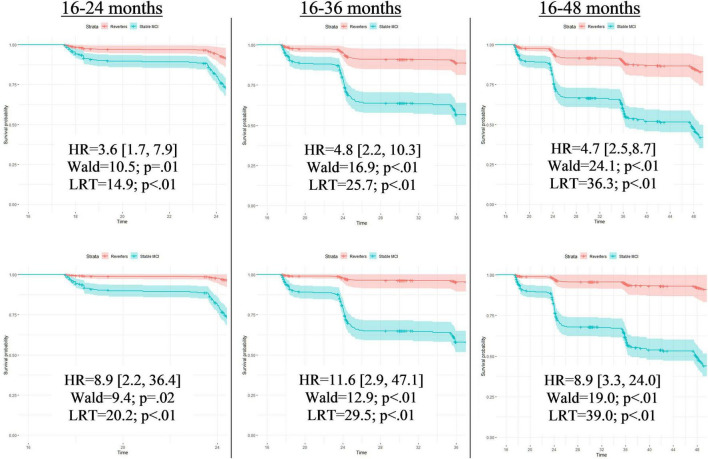
Full Cox proportional models for time until first dementia diagnosis by PE-unadjusted and PE-adjusted 12-month diagnoses. Cox proportional hazard models compared progression to dementia between those who were classified with mild cognitive impairment at follow-up (Stable MCI) and those who reverted to cognitively normal (Reverters). All models used classifications (Stable MCI vs. Reverter) as the independent variable of interest and months from baseline until first dementia diagnosis as the dependent variable. Covariates were age and education, fixed at the average level within the sample (age: 73.1 years; education: 16.4 years). Models in the top row display results completed with PE-unadjusted scores; models in the bottom row display results completed with the PE-adjusted scores. Each row designates the time frame for each model measured in months from baseline. Time frames were restricted to demonstrate how predictive the classification was for studies with various follow-up periods. As these hypothetical studies would not know if a participant converted to dementia past their follow-up period, those who converted after the endpoint of that specific model were censored (i.e., recoded as non-converters). Each model presents a hazard ratio (HR; [CI]) that indicates how much more likely the Stable MCI group was to convert to dementia compared to the Reverters. Wald tests and likelihood-ratio tests (LRT) are also included with associated *p*-values to denote the significance of the HR. The *Y*-axis of each of the 6 models provides the survival probability and the *X*-axis of each model provides the time frame until dementia conversion.

When false reverters were removed by adjusting for PEs, the median time until first dementia diagnosis was increased (+23 months). To further investigate this finding, we performed *post hoc* Cox proportional hazard models to compare progression to dementia from 12-month follow-up between those who were diagnosed as MCI at follow-up and those who reverted to CN. Across all models, the hazard ratio associated with increased risk of dementia progression among stable MCI participants was nearly twice as large when adjusted for PEs compared to PE-unadjusted diagnoses (average hazard ratio: PE-adjusted = 8.9, PE-unadjusted = 4.2; average percent increase = 110%). Figures 1, 2 displays hazard ratios and survival curves for all models. Supplementary Figure 1 provides additional Kaplan–Meier curves and risk tables for progression to dementia by diagnosis group.

There were 226 participants with baseline biomarker data. As shown in Table 6A, regardless of PE adjustment, approximately 70% of those who were diagnosed as MCI at follow-up were Aβ positive and 70% were P-tau positive at baseline. Similarly, regardless of PE adjustment, about 60% of reverters were Aβ positive and 45% were P-tau positive. There were 18 false reverters with biomarker data. The false reverter group had an Aβ positivity of 55% and a P-tau positivity of 40%. Table 6B displays the biomarker positivity rates for each classification group based on amyloid and P-tau positivity (i.e., A−/T−, A+/T−, A−/T+, and A+/T+). Regarding the false reverters, 72% (13/18) were positive for at least one biomarker.

**TABLE 6A:** Amyloid, total tau, and phosphorylated tau across classification groups.

	Full sample *N* = 226	Stable MCI		Reverters		False reverters *N* = 18
		Unadjusted *N* = 166	Adjusted *N* = 184	Unadjusted *N* = 60	Adjusted *N* = 42	
**Amyloid**						
Count	160	124	134	36	26	10
%	70.8%	74.7%	72.8%	60.0%	61.9%	55.6%
**T-tau**						
Count	123	101	106	22	17	5
%	54.4%	60.8%	57.6%	36.7%	40.5%	27.8%
**P-tau**						
Count	145	118	125	27	20	7
%	64.2%	71.1%	67.9%	45.0%	47.6%	39.9%

*Presents the number of participants (Count) and percent of sample (%) for three cerebrospinal fluid biomarkers: amyloid beta (Abeta), Tau, and phosphorylated tau (Ptau). Of the 329 participants, 226 had full biomarker data, which is presented in the “Full Sample” column. Participants were classified as “Stable MCI” if they retained their mild cognitive impairment (MCI) classification at follow-up; participants were classified as “Reverters” if they were classified as cognitively normal at follow-up. Classifications were made using practice effect-unadjusted (“Unadjusted”) and practice effect-adjusted (“Adjusted”) data. Those who were classified as MCI by the practice effect-adjusted data but not the unadjusted data are referred to as “False reverters.” The percent sample (%) was determined by dividing the number of biomarker-positive subjects in a cell by the total number of participants with that classification; e.g., 74% = 117/158.*

**TABLE 6B:** Combined amyloid and tau positivity profiles.

	Full	Stable MCI		Reverters		False
	Sample	Unadjusted	Adjusted	Unadjusted	Adjusted	Reverters *n* = 18
**A−T−**						
Count	39	22	27	17	12	5
Percent	17.3%	13.3%	14.7%	28.3%	28.6%	27.8%
**A + T−**						
Count	42	26	32	16	10	6
Percent	18.5%	15.7%	17.4%	26.7%	23.8%	33.3%
**A−T+**						
Count	27	20	23	7	4	3
Percent	11.9%	12.0%	12.5%	11.7%	9.5%	16.7%
**A + T+**						
Count	118	98	102	20	16	4
Percent	52.2%	59.0%	55.4%	33.3%	38.1%	22.2%
**A+ and/or T+**						
Count	187	144	157	43	30	13
Percent	82.7%	86.7%	85.3%	71.7%	71.4%	72.2%

*Presents the number of participants (Count) and percent of sample (%) for combinations of cerebrospinal fluid biomarker positivity: biomarker-negative (A−/T−), amyloid-positive and tau-negative (A+/T−), amyloid-negative and tau-positive (A−/T+), amyloid and tau positive (A+/T+), and positive for any biomarker (A+ and/or T+).*

## Discussion

The validity and utility of MCI criteria are weakened by high reversion rates, which have been a longstanding problem for MCI as a construct (Pandya et al., 2016). As a result, some practitioners are hesitant to use MCI as an early indicator of AD, despite the field’s goal of identifying and treating those on the AD trajectory as early as possible (Sperling R. A. et al., 2014; Canevelli et al., 2016; Pandya et al., 2016; Alexander et al., 2021). Among individuals in the ADNI sample who were diagnosed with MCI at baseline, adjusting for PEs led to a significant reduction in reversion to CN over 1 year (28.8% reduction in reversion rate). This meant that classifications were more stable across time, particularly for those with baseline amnestic MCI.

Pathologically, AD is characterized by a progressive change in amyloid beta and tau protein levels in the brain (Anand et al., 2017). Although there is conflicting evidence regarding the temporal staging of AD biomarkers and cognitive symptoms (Braak et al., 2011; Jack et al., 2013; Edmonds et al., 2015b; Veitch et al., 2019; Elman et al., 2020), it is likely that in most cases abnormal levels of amyloid beta are first reached, followed by abnormal levels of tau, which in turn affect cognition (Dubois et al., 2016; Jack et al., 2017, 2018). In our analyses, approximately half of the false reverters were amyloid positive while around a third were tau positive. Nearly three-quarters of the false reverters were positive for at least one of the two biomarkers. A comparison across all three groups – true reverters, false reverters, and stable MCI – suggests that the false reverters may be an intermediate/mixed biomarker group. Some of the false reverters who were biomarker negative (A−/T−) may have MCI that is unrelated to AD. However, it is also possible that even some of the false reverters who were biomarker negative may still be on the AD trajectory. We previously showed, for example, that after controlling for tau, cognitive function in A− individuals in the ADNI sample predicted progression to A+ status (Elman et al., 2020). Overall, the PE-adjustment reduced the number of reverters, resulting in more stable MCI diagnoses and may be identifying more people who are beginning to show clinically significant levels of AD biomarkers.

Use of a robust normal sample partially addresses PEs as the cut-off for MCI diagnosis varies at each timepoint based on the distribution of scores among participants who remain CN across all visits (Edmonds et al., 2015a; Eppig et al., 2017; Thomas et al., 2017, 2019). In a similar ADNI subsample, use of robust norms found a 1-year reversion rate of 15.8% (Thomas et al., 2019), which is similar to the rate found in the present study (17.3%). Whether the rates would be similar in different studies remains an open question. Using robust normal instead of normative data means that gauging impairment is based on what is a “super-normal” group that is, essentially, by definition, non-representative. This non-representativeness will be compounded further if the sample itself is not representative. For example, the robust normal group in ADNI is the highest functioning subgroup of what is already a very highly educated sample. In this approach there is no accounting for how PEs may be affecting classification into the robust normal group itself. It is possible that some individuals in that group might actually be classified as having MCI at some follow-up if their scores were adjusted for PEs at each time point based on a replacement participants approach. Moreover, PE estimation can be overestimated if attrition effects are not considered (Ronnlund et al., 2005; Elman et al., 2018). PEs based on a robust normal group may be inflated as compared to PEs within the overall sample because, by definition, this group does not have attrition (Eppig et al., 2017; Thomas et al., 2017). Finally, comparison of results from the present study with that of our prior study (Sanderson-Cimino et al., 2020) shows that it is important to differentiate the cognitive status of individuals at baseline because the magnitude of PEs differs for individuals who are CN at baseline versus those who have MCI at baseline.

Proponents of MCI as a diagnostic entity note that individuals with the diagnosis are more likely to progress to AD, and do so at a faster rate than CN individuals (Mitchell and Shiri-Feshki, 2009; Pandya et al., 2016). Those critical of MCI’s validity note that, while MCI is associated with AD, individuals with MCI are more likely to revert to CN over time than to progress to AD (Canevelli et al., 2016). Here we found that the false reverters progressed to dementia at approximately the same rate as individuals who were classified as MCI at both time points. In contrast, those who were classified as CN (i.e., true reverters) at follow-up progressed to dementia more slowly than the false reverters. These results are consistent with the notion that misclassification of these false reverters, caused by the failure to account for PEs, is weakening the predictive ability of MCI. This point is echoed by the time-to-dementia diagnosis of the reverter group. Removing the false reverters from the reverter group increased the time until first dementia diagnosis among those classified as CN by almost 2 years (37.28 versus 60.28 months).

Although adjusting for PEs slightly altered the median time until first dementia diagnosis, statistical comparisons between groups were non-significant. To further investigate these findings, we completed Cox proportional hazard models. Using PE-unadjusted data, we found that the stable MCI group converted to dementia significantly faster than the (false) reverter group, as expected. When models were completed with PE-adjusted data, we found that the hazard ratios sharply increased, suggesting that the PE-adjusted classifications improved differentiation between the (true) reverters and the stable MCI participants. Not accounting for PEs may thus obscure true effects or push significance above threshold, influencing subsequent interpretation.

Interestingly, hazard ratios were less different between PE-adjusted and PE-unadjusted models when analyses were completed over the full 150-month timeframe (HRs: 6.0. versus 3.7) compared to shorter time frames (24-month HRs: 8.9 versus 3.6; and 36-month HRs: 11.6 vs. 4.8). These results are consistent with the idea that PE adjustment leads to earlier detection of at-risk participants, which would be particularly important for studies with shorter follow-up periods. Importantly, clinical drug trials for AD typically involve shorter follow-up periods, so increasing the number of individuals expected to progress to dementia during the trial period will increase sensitivity to treatment effects. Therefore, failure to account for PEs may have a large impact on the design of treatment studies and interpretation of their results. Earlier detection of at-risk individuals is also of obvious importance for clinical care.

### Strengths and Limitations

All participants completed the logical memory test at a screening assessment, baseline, and 12-month visit; all other tests were completed only twice. Therefore, it is possible that the PE for logical memory is misestimated. However, as the effect size of the logical memory PE is similar to that of the other memory task (AVLT), it seems likely that our estimate is still valid.

Our time until dementia analyses did not account for death. Of the 329 participants included in these analyses, 33 passed away before study completion (10.0%). The modal time until death was 48-months past baseline visit (*n* = 8; 24% of deaths). Importantly, all participants who passed away were diagnosed as stable MCI (impaired at baseline and follow-up) by both the PE-adjusted and PE-unadjusted datasets. As such, although mortality may have impacted results, this effect was equal within the PE-adjusted and PE-unadjusted analyses.

The ADNI sample was not designed to be a population-representative study. It represents a population of older adults likely to volunteer for clinical trials, and consists primarily of white, highly educated individuals who may be at a higher genetic risk for dementia than typical Americans. Results of the present study may not be applicable to other studies with different sample characteristics or retest intervals. Additionally, age and education have been shown to impact PEs (Calamia et al., 2012; Gross et al., 2017). We strongly believe that the exact PE values found in this study should not be applied to other samples, particularly if they involve CN individuals with different demographics (i.e., age and education). However, a strength of the replacement-participants method of estimating PEs is that it is always tailored to the sample, including age and education, as well as the retest interval being studied. For example, in addition to the 1-year interval in the present study, the replacement-participants method has been used successfully in studies with intervals as long as 5–6 years (Ronnlund et al., 2005; Elman et al., 2018). Participant demographics and cognitive tests are always matched. Retest intervals may vary across studies, but PEs are calculated for the specific interval(s) used within a given study. Therefore, we explicitly recommend against using these PE estimates in other studies. Rather we encourage others to utilize the method within their study to more accurately generate PEs given their specific demographics, measures, and test–retest interval. The cost of including replacement participants might seem prohibitive, but it is actually a relatively small component in a large-scale study (Elman et al., 2018; Sanderson-Cimino et al., 2020). Elsewhere, we have shown that it could save millions of dollars in a large clinical trial because MCI is detected earlier, resulting in reductions in study duration and necessary sample size (Sanderson-Cimino et al., 2020). As shown in the present study, the method can be adapted to large studies that did not include replacements in their original design. However, building it into the original study design is clearly preferable.

## Conclusion

Here we have shown that a replacement method of PE adjustment significantly altered how we understand follow-up status in individuals who have already been diagnosed with MCI at the baseline assessment. Our results indicate that the replacement-participants method of adjustment for PEs results in fewer MCI cases reverting to CN, and improved predictability of progression to dementia. In sum, the results provide further support for the importance of accounting for PEs on cognitive tests in order to reduce misdiagnosis and increase earlier detection of progression to MCI or dementia.

## Alzheimer’s Disease Neuroimaging Initiative

Data collection and sharing for this project was funded by the Alzheimer’s Disease Neuroimaging Initiative (ADNI) (National Institutes of Health Grant U01 AG024904) and DOD ADNI (Department of Defense award number W81XWH-12-2-0012). ADNI was funded by the National Institute on Aging, the National Institute of Biomedical Imaging and Bioengineering, and through generous contributions from the following: AbbVie, Alzheimer’s Association; Alzheimer’s Drug Discovery Foundation; Araclon Biotech; BioClinica, Inc.; Biogen; Bristol-Myers Squibb Company; CereSpir, Inc.; Cogstate; Eisai Inc.; Elan Pharmaceuticals, Inc.; Eli Lilly and Company; EuroImmun; F. Hoffmann-La Roche Ltd. And its affiliated company Genentech, Inc.; Fujirebio; GE Healthcare; IXICO Ltd.; Janssen Alzheimer Immunotherapy Research & Development, LLC.; Johnson & Johnson Pharmaceutical Research & Development LLC.; Lumosity; Lundbeck; Merck & Co., Inc.; Meso Scale Diagnostics, LLC.; NeuroRx Research; Neurotrack Technologies; Novartis Pharmaceuticals Corporation; Pfizer Inc.; Piramal Imaging; Servier; Takeda Pharmaceutical Company; and Transition Therapeutics. The Canadian Institutes of Health Research is providing funds to support ADNI clinical sites in Canada. Private sector contributions are facilitated by the Foundation for the National Institutes of Health (www.fnih.org). The grantee organization is the Northern California Institute for Research and Education, and the study is coordinated by the Alzheimer’s Therapeutic Research Institute at the University of Southern California. ADNI data are disseminated by the Laboratory for Neuroimaging at the University of Southern California.

## Data Availability Statement

Publicly available datasets were analyzed in this study. This data can be found here: http://adni.loni.usc.edu/.

## Ethics Statement

The studies involving human participants were reviewed and approved by University of California, San Diego. Written informed consent for participation was not required for this study in accordance with the national legislation and the institutional requirements.

## Author Contributions

MS-C and WK conceived the study. XT and AG provided guidance on statistical analysis. EE, MB, JSE, and KT made determination of MCI diagnoses. MS-C, WK, JAE, MP, and DG contributed to the practice effects methodology. WK, CF, ML, and MS-C obtained primary funding to support this work. All authors provided critical review and commentary on the manuscript.

## Conflict of Interest

MB receives royalties from Oxford University Press. The remaining authors declare that the research was conducted in the absence of any commercial or financial relationships that could be construed as a potential conflict of interest.

## Publisher’s Note

All claims expressed in this article are solely those of the authors and do not necessarily represent those of their affiliated organizations, or those of the publisher, the editors and the reviewers. Any product that may be evaluated in this article, or claim that may be made by its manufacturer, is not guaranteed or endorsed by the publisher.

## Footnotes

1
adni.loni.usc.edu

2
http://adni.loni.usc.edu/methods
