# Supplementary figures and images for: Practice Effects in Mild Cognitive Impairment Increase Reversion Rates and Delay Detection of New Impairments
Source: Front Aging Neurosci. 2022 Apr 25;14:847315. doi: 10.3389/fnagi.2022.847315 (PMC9083463; doi:10.3389/fnagi.2022.847315)

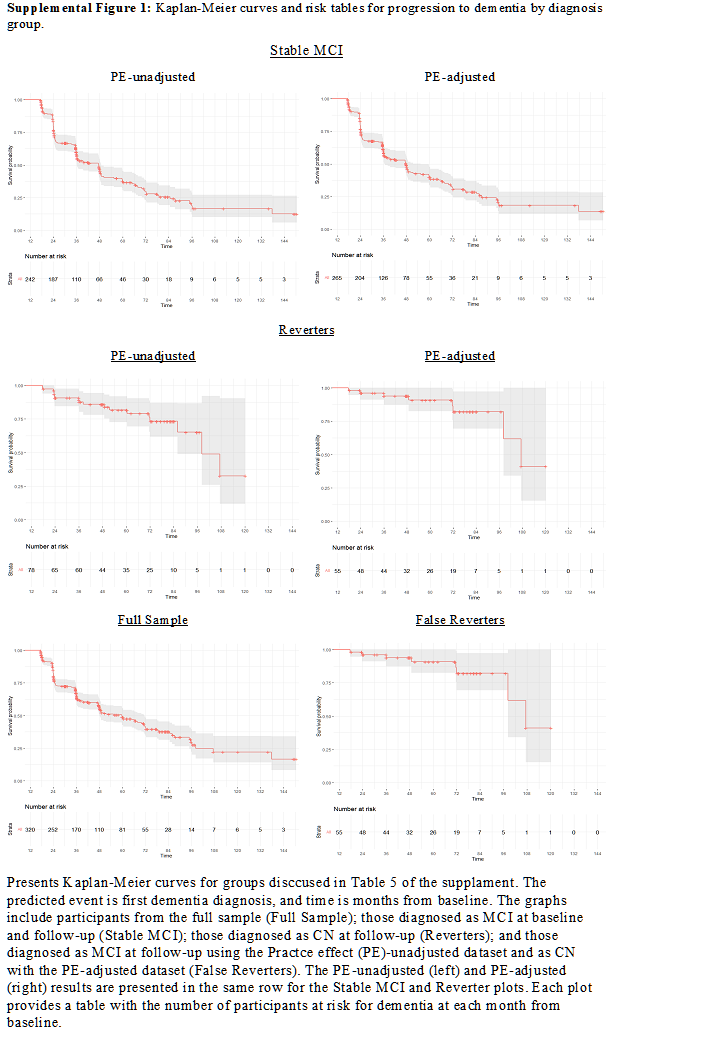

Supplement: Supplementary file 1 [file Figure_1.TIF]
